# New Mode of Reducing Exomphalus

**Published:** 1844-09-21

**Authors:** 


NEW MODE OF REDUCING EXOMPHALUS.

Dr. Pasquier was called to see a patient affected with strangulated hernia umbilicalis, against which every attempt at reduction had failed; and the symptoms increased, in intensity, to such an exteut, that notwithstanding the danger, the operation seemed indispensable. In this dilemma, Dr. P. succeeded by the following novel expedient:—he seized the parietes of the abdomen, above and below the umbilicus, raised the patient, and gave him several jerks. The hernia disappeared, and with it the dangerous symptoms. This method ought never to be had recourse to, but in recent cases, because, if the strangulation had existed some time, the pressure of the stricture on the intestine might have so weakened it as to cause it to burst.—*Ibid.*

